# Predictive factor and antihypertensive usage of tyrosine kinase inhibitor-induced hypertension in kidney cancer patients

**DOI:** 10.3892/ol.2014.2060

**Published:** 2014-04-11

**Authors:** KOUJI IZUMI, SHINGO ITAI, YOSHIKO TAKAHASHI, AERKEN MAOLAKE, MIKIO NAMIKI

**Affiliations:** 1 Department of Integrative Cancer Therapy and Urology, Kanazawa University Graduate School of Medical Science, Kanazawa, Ishikawa 920-8641, Japan; 2 Department of Medicinal Informatics, Kanazawa University Graduate School of Medical Science, Kanazawa, Ishikawa 920-8641, Japan

**Keywords:** hypertension, kidney cancer, tyrosine kinase inhibitor

## Abstract

Hypertension (HT) is the common adverse event associated with vascular endothelial growth factor receptor-tyrosine kinase inhibitors (VEGFR-TKI). The present study was performed to identify the predictive factors of TKI-induced HT and to determine the classes of antihypertensive agents (AHTA) that demonstrate optimal efficacy against this type of HT. The charts of 50 cases of patients that had received VEGFR-TKI treatment were retrospectively examined. The association between patient background and TKI-induced HT, and the effect of administering AHTA were analyzed. High systolic blood pressure at baseline was identified to be a predictive factor for HT. In addition, there was no difference observed between calcium channel blockers (CCBs) and angiotensin receptor II blockers (ARBs) as first-line AHTA for the control of HT. The findings of the present study may aid with predicting the onset of TKI-induced HT, as well as for its management via the primary use of either CCBs or ARBs.

## Introduction

It had been indicated that antitumor agents do not have a beneficial effect on patient survival in cases of kidney cancer. Only cytokine therapies, including interferon-α and interleukin-2, have been used for patients with metastatic (or surgically unresectable) kidney cancer (MKC). However, these agents demonstrate insufficient efficacy (1–3). Subsequent to the phase III trial investigating the effects of sorafenib on MKC, the vascular endothelial growth factor (VEGF) signaling pathway inhibitors have emerged as leading treatments for MKC (4–8). Three VEGF receptor-tyrosine kinase inhibitors (VEGFR-TKI), sorafenib, sunitinib and axitinib, are available for patients with MKC in Japan, as of 2012. The affinity and selectivity of VEGFR-TKI for VEGFR are varied, and accordingly the incidence and severity of adverse events (AE) also differ (5–7). Hypertension (HT) is the most common AE associated with VEGFR-TKI therapy and it occasionally becomes a critical factor for the discontinuation of the treatment (5–8). By contrast, the onset of HT following the initiation of VEGFR-TKI treatment has been reported as a possible biomarker of a good response to VEGFR-TKI (9). Therefore, the control of HT is extremely significant for the continued use of VEGFR-TKI and to achieve the optimal outcome in MKC treatment. The present study was performed to identify the predictive factors of VEGFR-TKI-induced HT, and to determine the classes of antihypertensive agents (AHTAs) that demonstrate the optimum efficacy against secondary HT.

## Patients and methods

### Study population

All studies were performed retrospectively in Kanazawa University (Kanazawa, Japan) using the charts of patients who were hospitalized at the Department of Urology. Patients with MKC who underwent VEGFR-TKI (sorafenib, sunitinib and axitinib) therapy were analyzed. The AHTAs that were administered were categorized according to their mechanisms of action. The study was in accordance with the Declaration of Helsinki Guidelines.

### Definition of HT

HT was defined as systolic blood pressure (BP) of >140 mmHg, corresponding to Grade 2 of the National Cancer Institute-Common Terminology Criteria for Adverse Events version 4.0 (10). The policy of the Department of Integrative Cancer Therapy and Urology, Kanazawa University Graduate School of Medical Science (Kanazawa, Japan) for commencing AHTA administration was also the same as the definition of HT. The BP of all cases was reviewed prior to VEGFR-TKI administration (baseline), between the onset of *de novo* HT and commencing AHTA administration, and on HT improvement following AHTA administration. The average BP levels at identical times on three consecutive days were calculated and used for analyses; however, single BP measurements were also used if the patient was discharged and became an outpatient.

### Statistical analysis

Statistical analyses were performed using commercially available software (Prism; GraphPad Software, Inc., San Diego, CA, USA). Comparisons between two groups were performed by unpaired two-sided t-test, Fisher’s exact test and χ^2^ test to identify trends. The probability of administrating AHTA was estimated using the Kaplan-Meier method. In all analyses, P<0.05 was considered to indicate a statistically significant difference.

## Results

### Patient characteristics

**Figure 1:**
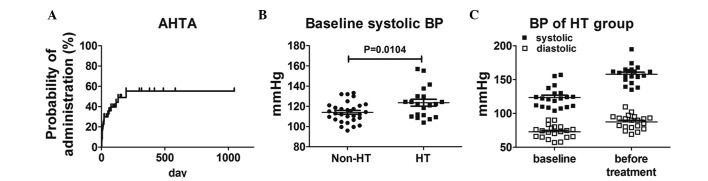
(A) Probability of administrating AHTA is shown using the Kaplan-Meier method. (B) Average systolic BP at baseline in non-HT (n=30) and HT (n=20) groups. (C) Average systolic and diastolic BP at baseline and before AHTA administration in the HT group. AHTA, antihypertensive agents; BP, blood pressure; HT, hypertension.

In total, 50 VEGFR-TKI administration events from 41 patients were analyzed and the patient demographic data are shown in Table I. The number of cases of sorafenib, sunitinib and axitinib administration were 18, 27 and 5, respectively. A total of 22 patients had pre-existing HT, and one or two AHTA had previously been prescribed. The probability of AHTA administration is shown in Fig. 1A.

### Predictive factor of VEGFR-TKI-induced HT

Of the 50 cases, 20 had HT subsequent to VEGFR-TKI administration, and their backgrounds were compared with the 30 non-HT cases (Table II). The median systolic BP at baseline was significantly higher in 20 HT cases (P=0.0104), and the distributions of systolic BP in the non-HT and HT groups are shown in Fig. 1B. In total, 2 cases exhibited Grade 2 HT at baseline and commenced AHTA following the deterioration of HT to Grade 3 (systolic BP, >160 mmHg). The distributions of BP at baseline and prior to AHTA administration in the HT group are also shown in Fig. 1C.

### Administration of AHTA

**Figure 2:**
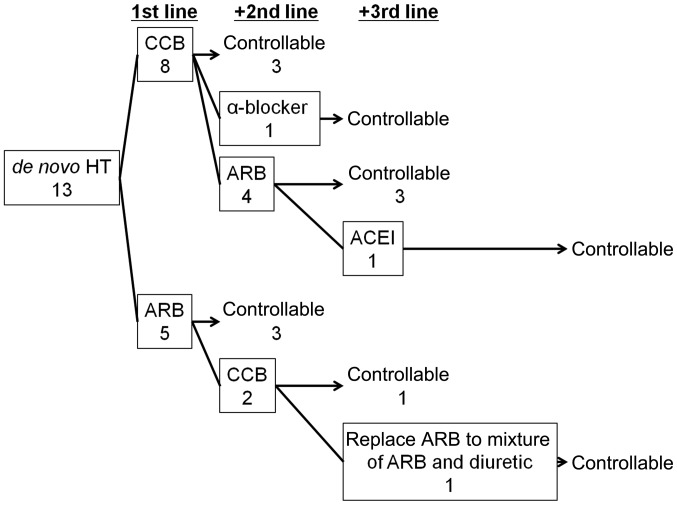
AHTA administered for EGFR-TKI-induced *de novo* HT are shown. CCB or ARB was administered for *de novo* HT as a first-line therapy, and second- and third-line AHTA were added if necessary. No significant difference was identified between the control rate of CCB and ARB as first-line treatments (P=0.5921). AHTA, antihypertensive agents; EGFR-TKI, epidermal growth factor receptor-tyrosine kinase inhibitor; HT, hypertension; CCB, calcium channel blocker; ARB, angiotensin receptor II blocker; ACEI, angiotensin-converting enzyme inhibitor.

The variations in efficacy, between AHTA administration in 13 cases of *de novo* HT and no AHTA administration prior to initiation of VEGFR-TKI therapy, were analyzed. The first-line AHTA treatment was either calcium channel blockers (CCBs) or angiotensin receptor II blockers (ARBs). There was no significant difference identified between the control rate of CCB and ARB as first-line treatments (3/8 for CCB treatment and 3/5 for ARB, P=0.5921; Fig. 2).

## Discussion

It is important to identify the predictive factors for key AE that are associated with VEGF-TKI to prevent treatment discontinuation, as well as to predict the population that may show a good response to these agents. Furthermore, it may contribute to an improved outcome. In a study using axitinib treatment for Japanese patients with MKC, Tomita *et al* (11) indicated that baseline proteinuria and soluble VEGFR-2 levels may be predictive factors of axitinib-induced proteinuria, which may also be a predictive factor of a good response to axitinib. With regard to HT, a study of the VEGFR-TKI, cediranib, for non-small cell lung cancer indicated that predictors of VEGFR-TKI-induced HT were as follows: Eastern Cooperative Oncology Group performance status 0; female; normal lactate dehydrogenase levels; and no prior peripheral vascular disease (12). A meta-analysis of sunitinib indicated a significantly higher incidence of sunitinib-induced HT in MKC compared with gastrointestinal stromal tumors (13). These studies indicated that predictors of VEGFR-TKI-induced HT in patients with MKC should exist, and should be identified for extended VEGFR-TKI use during MKC treatment. In the present study, a high baseline systolic BP was the only predictive factor of VEGFR-TKI-induced HT. This result is reasonable and indicated that the evaluation of BP at baseline is significant for managing VEGFR-TKI administration. As controversy remains regarding the optimal treatment for VEGFR-TKI-induced HT, the category of AHTA that is preferable for treatment of secondary HT, based on the charts of 13 *de novo* HT cases, was investigated. As expected, two major categories of AHTA were used as first-line treatments for VEGFR-TKI-induced HT, CCB and ARB, and there was no difference in efficacy identified between these two AHTA categories. Although certain review studies proposed the use of AHTA for VEGFR-TKI-induced HT, there is no evidence that the specific usage of AHTA is a requirement for VEGFR-TKI-induced HT (14–16). However, the unique situation of VEGFR-TKI-induced HT should be considered. It has been reported that ARB may have antitumor effects due to the inhibition of angiotensin II signaling (17). A systematic review indicated that ARB improved progression-free survival in patients with MKC, and that ARB administration was protective against prostate-specific antigen failure in patients with prostate cancer (18). In addition, ARB decreased pressure in the glomerulus, and reduced proteinuria, which consequently inhibited the deterioration of renal function (19,20). As proteinuria is a critical AE of VEGFR-TKI and HT (7,11), ARB may be preferable for patients that are treated with VEGFR-TKI. By contrast, ARB cannot be used for patients with bilateral renal artery stenosis or solitary kidney associated with renal artery stenosis, or for patients with an elevated creatinine level (>2.0 mg/dl), therefore, CCB may be appropriate in such cases. Although this was a retrospective study with a small sample size, it was shown that baseline BP may predict VEGFR-TKI-induced HT. Furthermore, no difference in efficacy was identified between CCB and ARB for VEGFR-TKI-induced HT. These findings may aid clinicians with predicting the onset of VEGFR-TKI-induced HT and for its management via the primary use of CCB or ARB.

## Figures and Tables

**Table I:** Patient demographics.

Demographic	Value
Number	50
Median age, year	65 (26–85)
Gender	
Male	43
Female	7
Prior nephrectomy	
Yes	30
No	20
TKI	
Sorafenib	18
Sunitinib	27
Axitinib	5
Median TKI administration days	102 (7–1117)
Median initial BP	
Systolic	116 (96–157)
Diastolic	72 (57–90)
Number of prior AHTA	
0	28
1	13
2	9
Prior AHTA	
CCB	17
ARB	9^a^
ACEI	1
Others	4
TKI-induced HT	
Yes	20
No	30

a One mixture of ARB and diuretic was included.

Values in parentheses indicate the range. TKI, tyrosine kinase inhibitor; BP, blood pressure; AHTA, antihypertensive agents; CCB, calcium channel blocker; ARB, angiotensin receptor II blocker; ACEI, angiotensin converting enzyme inhibitors; HT, hypertension.

**Table II:** Comparison of the backgrounds between HT and non-HT patients.

Background	Non-HT	HT	P-value
Number	30	20	
Median age, year	65 (26–80)	66 (47–85)	0.5992
Gender			
Male	26	17	1.0000
Female	4	3	
Prior nephrectomy			
Yes	17	13	0.7688
No	13	7	
TKI			
Sorafenib	11	7	0.9923
Sunitinib	16	11	
Axitinib	3	2	
Median TKI administration days	69 (5–1047)	188 (21–1117)	0.1895
Median initial BP			
Systolic	114 (96–133)	122 (104–157)	0.0104
Diastolic	70 (58–83)	74 (57–90)	0.2555
Number of prior AHTA			
0	15	13	0.3486
1	10	3	
2	5	4	
Administered AHTA			
CCB	13	4	0.3127
ARB	5^a^	4	
ACEI	0	1	
Others	2	2	

a One mixture of ARB and diuretic was included.

Values in parentheses indicate the range. HT, hypertension; TKI, tyrosine kinase inhibitor; BP, blood pressure; AHTA, antihypertensive agents; CCB, calcium channel blocker; ARB, angiotensin receptor II blocker; ACEI, angiotensin converting enzyme inhibitors.
